# Stroke awareness among Dubai emergency medical service staff and impact of an educational intervention

**DOI:** 10.1186/s13104-017-2585-x

**Published:** 2017-07-06

**Authors:** Fatima Shire, Zahra Kasim, Suhail Alrukn, Maria Khan

**Affiliations:** 1 Department of Emergency Medicine, Rashid Hospital, Dubai, UAE; 2 Department of Neurology, Rashid Hospital, Dubai, UAE

**Keywords:** Stroke, Prehospital care, Education, Thrombolysis

## Abstract

**Background:**

Emergency medical services (EMS) play a vital role in expediting hospital arrival in stroke patients. The objective of our study was to assess the level of awareness regarding pre-hospital identification and management of acute stroke among EMS Staff in Dubai and to evaluate the impact of an educational lecture on their knowledge.

**Methods:**

Ours was a cross-sectional study with a pre-test and post-test design. The intervention was an educational lecture, based on the updated guidelines in pre-hospital care of acute stroke. Participants were assessed before and after the intervention on various aspects of stroke care. Paired t test were used to compare the impact of the intervention.

**Results:**

A total of 274 EMS workers participated in our study. The baseline knowledge of participants regarding stroke types was inadequate with only 68% correctly identifying these. 79% were able to name the cardinal stroke symptoms. Knowledge of stroke mimics was poor with only 6.6% identifying stroke mimics correctly. With respect to management, most participants were unable to correctly identify the points to illicit in the history of an acute stroke patient (25.2%) and also the steps in pre-hospital management (40%). All these aspects showed remarkable improvement post intervention.

**Conclusion:**

The baseline awareness of most aspects of acute stroke identification and management was poor in our EMS participants. Our educational lecture proved effective in improving this knowledge when tested immediately post intervention. However, there is a need to re-assess this at periodic intervals to identify the need for refresher courses on pre-hospital stroke management.

**Electronic supplementary material:**

The online version of this article (doi:10.1186/s13104-017-2585-x) contains supplementary material, which is available to authorized users.

## Background

Stroke is a common neurological disorder and the leading cause of morbidity and mortality worldwide. According to the Global burden of disease study, in 2013 there were 6.5 million deaths from stroke, 25.7 million stroke survivors and 10.3 million new strokes globally [1]. This burden is expected to rise in future with cerebrovascular disease expected to become the fourth commonest cause of ongoing disease burden worldwide by the year 2020 [2].

United Arab Emirates is no exception. As the country undergoes economic transition, there is an increase in non-communicable diseases like diabetes and obesity [3, 4]. Although prevalence data for stroke is not yet available from the country, a health statistic done by the Health Authority of Abu Dhabi (HAAD) in 2014 identified circulatory disorders (stroke and ischemic heart disease) as the leading cause of mortality amounting to 37% of all causes [5].

Ischemic strokes constitute the vast majority of strokes and tissue plasminogen activator (t-PA) is the only approved treatment worldwide for treating these stroke. According to American Heart Association and American Stroke Association (AHA/ASA) recommendations the goal of treatment is to restore blood flow to the affected area of the brain as quickly as possible within the first 3 h by administering intravenous thrombolytic therapy (t-PA) [6]. This window can be extended to 4.5 h in selected patients [7]. More recently, endovascular therapy has also been approved for patients with large anterior circulation occlusions up to 6 h of stroke onset. All these therapies are time dependent and hence the need for early identification and prompt transport to stroke centers is of paramount importance.

However, in most regions of the world t-PA continues to be underutilized. A major reason behind this is late presentation of stroke patients to appropriate facility for thrombolysis [8]. Emergency medical services (EMS) play a critical role in reducing pre-hospital delays and ensuring timely administration of intravenous thrombolysis. This is because these paramedical staff are the first medical contact for a large majority of stroke patients [9]. Furthermore, use of EMS has been shown to reduce time from onset of stroke to emergency room arrival as well as time from arrival to brain imaging [10–12].

American Heart association (AHA) recommends that for EMS the primary goals are rapid assessment, early stabilization and rapid transport to proper specialized hospitals [6]. However, a number of studies have shown that paramedical staff lack proper knowledge of stroke and miss the diagnosis leading to treatment delays. Several interventional studies have been done to improve the knowledge of EMS staff with regard to stroke symptoms and its pre-hospital management [9, 13]. These studies have been shown to improve prehospital stroke diagnosis, and reduce delays to treatment.

Rashid Hospital, Dubai is one of the largest centers in the Middle East region providing thrombolysis. Ever since the establishment of a stroke service at Rashid Hospital in 2012, the number of patients receiving thrombolysis annually is on the rise. The hospital admits an average of 500 ischemic stroke patients every year and around 13% of these receive intravenous thrombolysis. There is now mechanical thrombectomy also available, and so far around 40 patients have benefited from this service. The city has a well-established EMS that is utilized by a large majority of patients presenting to the hospital.

As the stroke services expand in the city, it is important to improve the pre-hospital care of stroke patients. The objectives of our study were to assess the level of awareness regarding pre-hospital identification and management of acute stroke among EMS Staff in Dubai and to evaluate the impact of an educational lecture on their knowledge.

## Methods

### Study setting

This study was conceived and carried out by the Stroke Service at Rashid Hospital, Dubai. Rashid Hospital’s stroke unit is the only dedicated stroke unit in Dubai. The hospital admits more than 45 cases per month and provides round the clock services for stroke care including intravenous thrombolysis since the establishment of its stroke services in 2012.

The Emergency medical service (EMS) staff were recruited from the Dubai Corporation for Ambulance Services (DCAS), which serves the entire city of Dubai. DCAS has 68 stations distributed geographically within the Emirate of Dubai, and employs over 800 EMS staff [14].

### Study design

This was an interventional study with a pre-test and post-test design. The intervention was an educational lecture based on the AHA/ASA guidelines in pre-hospital care of acute stroke [6]. For evaluating knowledge, we designed an open ended questionnaire covering all aspects of pre-hospital stroke assessment and care.

### Study timeline and sampling

The study was conducted between 8th and 21st of April 2014 in Dubai Ambulance Center. Sampling technique was convenience sampling. We selected EMS staff based on their availability and willingness to participate in the study. Staff members who were working in the private sector were excluded even though they were affiliated with DCAS.

### Intervention

The participants were grouped into two based on their availability. The questionnaire was administered to each group before the intervention to assess their baseline knowledge. This was followed by the intervention in the form of an lecture that covered stroke etiology, symptoms, pre-hospital assessment and management and therapeutic window of thrombolytic administration in ischemic stroke. The lecture used a PowerPoint presentation, was prepared based on AHA guidelines [6] and was delivered by a stroke neurologist. The session was interactive and allowed participants to clarify any ambiguities they had. The questionnaire was re-administered, the same day as post-test to evaluate the improvement in their knowledge.

### Data collection tool

The tool used for pre and post intervention assessment was a paper based questionnaire. The main input for the questionnaire came from SA who is a stroke neurologist. It consisted of four sections with multiple choice and closed ended questions; demographic data, knowledge about stroke and its clinical assessment, pre-hospital Management and knowledge about thrombolytic therapy. Every correct answer was scored including multiple choice questions. Participants were scored based on number of correct answers in both pre and post intervention. The total score for the entire assessment was 27. All areas left blank were counted as zero and considered as a lack of knowledge in that area. (The data collection tool is provided in Additional files 1, 2).

Pre-testing of the questionnaire was done by administering it to a 10 year one residents from emergency department at Rashid Hospital. Based on their feedback, modifications were made to improve clarity.

### Data analysis

All data were entered in Microsoft Excel and transferred to SPSS v.20 for analysis. We report mean ± standard deviations for all continuous variables. For categorical variables, percentages are reported. The pre and post-intervention scores were compared using paired sample t test. These results are reported as mean scores, difference in scores and their 95% confidence intervals. p value of <0.05 was considered significant.

## Results

A total of 274 EMS workers participated in our study. Mean age of the participants was 32.88 years (SD 6.29 years). Three quarters of the participants were males and almost half of them were of South Asian origin. Most of the workers had more than 5 years of experience and 76% reported seeing 1–4 stroke cases per month. Only 37% reported attending any educational courses on stroke. Table 1 shows the basic demographic data for the participants.

**Table 1:** Demographic and other characteristics of EMS participants n = 274

Demographic and other characteristics	
Age (years) mean ± standard deviation	32.88 ± 6.29
Range (years)	24–59

	n (%)
Gender (n = 267)^a^	
Male	201 (75.3)
Female	66 (24.7)
Ethnicity (n = 251)^a^	
UAE Arab	9 (3.6)
Non UAE Arab	20 (8.0)
Caucasian	12 (4.8)
South Asian	117 (46.6)
Far East Asian	90 (13.6)
African	3 (1.2)
Years of experience (n = 264)^a^	
Less than 1 year	52 (19.7)
1–3 years	48 (18.2)
3–5 years	35 (13.3)
More than 5 years	129 (48.9)
Attended educational course on stroke (n = 262)^a^	97 (37.0)
Number of stroke cases attended to per month (n = 260)^a^	
None	24 (9.2)
1–4	198 (76.2)
5–10	23 (8.8)
Greater than 10	15 (5.8)

*UAE* United Arab Emirates

^a^ Number in brackets represents the number of people who answered the question

Only 68% of the participants were able to correctly identify the two main stroke types. A higher percentage (78%) could identify three cardinal stroke symptoms on baseline evaluation. Knowledge of stroke mimics was deficient with only 6.6% identifying stroke mimics correctly. Post intervention, an improvement was seen in all domains of stroke knowledge. In particular, the knowledge on stroke mimics improved to 88.3% (Table 2).

**Table 2:** Performance in individual questions

Question	Pre-intervention % correct response	Post-intervention % correct response
Section A: stroke knowledge and assessment		
Correctly identified 2 stroke types	186 (67.9)	268 (97.8)
Correctly identified 3 cardinal symptoms	216 (78.8)	268 (97.8)
Identified scale for stroke identification	169 (61.7)	255 (93.1)
Correctly identified 3 stroke mimics	18 (6.6)	242 (88.3)
Section B: pre-hospital management		
Correctly identified triage category for acute stroke	206 (75.2)	247 (90.1)
Correctly identified 3 points to illicit in history	69 (25.2)	211 (77.0)
Correctly identified 5 management steps for acute stroke	40 (14.6)	187 (68.2)
Correctly identified destination for acute stroke cases	233 (85.0)	251 (91.6)
Will pre-notify hospital for acute stroke arrival	256 (93.4)	267 (97.4)
Section C: knowledge of thrombolysis		
Had knowledge of TPA	225 (82.1)	265 (96.7)
Had knowledge of window for thrombolysis	0 (0)	239 (87.2)
Correctly identified 4 contraindications to TPA	25 (9.1)	193 (70.4)
Correctly identified the major complication of using TPA	175 (63.9)	256 (93.4)

Only a quarter of the participants could correctly identify three points to illicit in history of acute stroke patient, and only 40% knew the proper steps in pre-hospital management. Both these aspects showed remarkable improvement post intervention (77% and 68.2 respectively).

Most EMS participants had some idea of t-PA, but none could identify correctly the window period for thrombolysis. Only 9% knew of the contraindications for intravenous thrombolysis. Post intervention 87.2% correctly identified the window for thrombolytic therapy and 70% could name the contraindications for the drug.

We compared the pre and post education scores overall and for each section individually. The mean overall score improved by 7.53 points after the education intervention (95% CI 7.06–8.00, p < 0.001). The scores improved significantly in individual sections also with the greatest difference being observed in the section on stroke knowledge and assessment (Table 3).

**Table 3:** Pre and post intervention scores

Score	Pre-intervention	Post-intervention	Difference (95% CI)	p value
Total score (max 27)	17.29 (4.73)	24.81 (3.91)	7.53 (7.06–8.00)	<0.001
Section A score (max 9)	5.76 (1.79)	8.65 (1.28)	2.89 (2.68–3.09)	<0.001
Section B score (max 11)	7.63 (2.16)	9.87 (1.85)	2.24 (2.00–2.48)	<0.001
Section C score (max 7)	3.90 (2.08)	6.30 (1.28)	2.40 (2.17–2.63)	<0.001

All scores are given as means (standard deviation)

Section A: stroke knowledge and assessment

Section B: pre-hospital management

Section C: knowledge of thrombolysis

We evaluated also whether there was a difference in baseline knowledge of participants based on the number of stroke cases they were seeing monthly and their years of experience. No difference was found in the knowledge between those seeing less than 5 cases versus those seeing 5 or more cases per month. However, differences existed depending on years of experience of the EMS staff. Participants with less than 5 years of experience were significantly more knowledgeable regarding stroke symptoms (84% vs 73%, p value 0.04), stroke scales (73.1% vs 48.8%, p value <0.001), and awareness of thrombolytic therapy (88.3% vs 75.2%, p value 0.005). No significant differences were observed in the other domains.

## Discussion

Emergency medical services play a critical role in timely management of acute ischemic stroke patients. Correct identification of stroke cases by EMS staff not only results in expedited transfer to appropriate facility, it has also been shown to reduce in-hospital delays and a greater odds of receiving thrombolysis [15, 16].

Our study was conducted with this objective in mind. The overall awareness amongst the general population regarding stroke and its risk factors and presentation is severely deficient in the Arab world [17]. Therefore an even greater responsibility rests on the shoulders of EMS staff to promptly identify and expedite hospital transfers. Any initiative to improve stroke recognition and early stabilization of stroke victims by EMS staff is bound to have an impact on the overall quality of acute stroke care.

Our results demonstrate that the baseline knowledge of our EMS staff with regard to most aspects of acute stroke identification and management needs improvement. None of the 274 participants were able to identify the correct therapeutic window for intravenous thrombolysis, and very few were aware of its contraindications. Our educational intervention was effective in improving their knowledge especially about stroke mimics, pre-hospital management and therapeutic window period for thrombolytic therapy.

A study conducted in Taiwan [18] demonstrated a similar improvement in knowledge amongst EMS staff when tested immediately post intervention and also after 3 months. Their EMS staff was also deficient in knowledge at baseline although 48% had previously attended an educational session on acute stroke management, a proportion slightly higher than ours (37%). Brey et al. from Australia [19] employed a similar educational intervention to improve knowledge amongst their paramedics. The questions regarding stroke signs and symptoms were answered correctly by most of the participants in this study, unlike our EMS staff. However, their knowledge regarding thrombolytic therapy and its correct time window was deficient as was the case with our participants. The authors demonstrated that the sensitivity of paramedic diagnosis of stroke improved to 94% post intervention.

Knowledge regarding stroke symptoms was shown to be good in a national pre-hospital stroke survey done in the United States, however, the knowledge of correct therapeutic window for thrombolysis was poor [11]. A more recent study from Saudi Arabia also demonstrates similar findings [20] and to the best of our knowledge is the only other study of this kind from the Middle East region. Our findings are consistent with results from these studies.

A review by Fassbender et al. [21] highlights the importance of pre-hospital stroke management to reduce treatment delays. Education of EMS staff to ensure correct identification of stroke victims, use of standardized tools for symptom recognition and pre-notifying hospitals to receive patients have all proven effective in reducing time delays [22, 23] and also in improving rates of thrombolysis [24]. Most of such interventional studies have been done in the West, and very little data are available from the Middle East region on the effect of such educational lectures on the paramedics’ knowledge in this region.

Also, a number of these studies have focused on stroke recognition alone, without much focus on the therapeutic window for thrombolysis. We demonstrated that our intervention was effective in improving the knowledge of our EMS staff. Studies have also shown that there is a discrepancy between knowledge of stroke and reaction in real time situations [25]. Although we did not evaluate the impact of this knowledge change in our study in terms of stroke recognition and thrombolysis rates, we believe that this intervention will translate into better identification and management of stroke cases.

We also found that paramedics who are relatively junior professionals with experience less than 5 years were more knowledgeable regarding stroke symptoms, scales and thrombolysis compared to their senior colleagues. One possible explanation for this difference can be the more recent training of these junior staff. With the increasing burden of stroke in the region, and also the availability of thrombolysis, it is possible that training programs are focusing on this aspect as well.

Our study is the first of its kind to be done in the United Arab Emirates. We have shown that our EMS staff are deficient in knowledge of stroke and its pre-hospital management. However, a significant improvement in scores following our educational lecture, suggests that this simple intervention can be scaled up to improve pre-hospital care of stroke patients. Another strength of our study is the before and after design, that took care of potential confounders that we did not measure.

### Limitations

Our study has certain limitations. Firstly, it assessed only the immediate impact of intervention. How long these paramedics will retain this knowledge is not known. Secondly, whether this improvement in knowledge will translate into better rates of thrombolysis and shorter pre-hospital delays also needs to be studied. Thirdly, the Intervention was delivered in English and assessments were also done in the same language. In a multi-ethnic multi-linguistic city, where our study participants were also of varied nationalities, this might have served as a limitation to their understanding, which we did not assess objectively. However, English remains the main mode of communication in the region due to this diversity.

## Conclusion

Knowledge of stroke mimics, pre-hospital management and appropriate window for thrombolytic therapy were lacking in a majority of the EMS staff in Dubai. Our educational intervention was effective in improving their level of knowledge when assessed immediately post intervention.

Our study shows that such educational lectures will be effective in improving knowledge of EMS staff with regard to acute stroke and its pre-hospital management. We recommend carrying out further studies to evaluate the need for refresher courses and to assess if these lectures are effective in reducing pre hospital delays and improving thrombolysis rates. We also recommend developing websites that provide training opportunities to the EMS staff in the form of presentations and lectures.

## Additional files

**Additional file 1.** Data collection Form. The form on which the EMS staff were evaluated, pre and post intervention.

**Additional file 2.** Key to the Data collection Form. The answer key which was pre-prepared for marking the pre and post test forms.

## Abbreviations

UAE: United Arab Emirates
EMS: emergency medical services
t-PA: tissue plasminogen activator
AHA/ASA: American Heart Association/American Stroke Association
DCAS: Dubai Corporation for Ambulance Services
