# Supplementary material for: Stroke awareness among Dubai emergency medical service staff and impact of an educational intervention
Source: BMC Res Notes. 2017 Jul 6;10:255. doi: 10.1186/s13104-017-2585-x (PMC5500952; doi:10.1186/s13104-017-2585-x)
Supplement: Supplementary file 1 — Additional file 1. Data collection Form. The form on which the EMS staff were evaluated, pre and post intervention. [file 13104_2017_2585_MOESM1_ESM.docx]

**Study Title: Assessment of knowledge of EMS staffs regarding acute stroke in Dubai**

You are being invited to participate in a research study through answering a questionnaire. There are no known risks if you decide to participate in this research study. There are no costs to you for participating in the study.

I have read the Consent Form and conditions of this study. I have had all my questions answered. I hereby acknowledge the above and give my voluntary consent

Signature:

1. ***Demographic data:***

- Age:
- Sex:
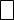
 Male
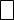
 Female
- Nationality:
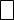
UAE
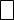
 Arab
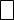
 Caucasian
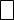
 Indian subcontinent
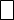
 Far East
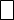
 African
- Years of experience in EMS field?
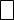
 Less than1 year
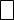
1-3 years
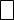
3-5 years
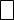
 more than 5 years
- Does your hospital have a stroke program?
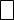
 Yes
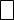
 No
- Have you get an educational course about stroke in the last 2years?
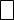
 Yes
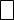
 No
- How frequent you see stroke victim per month?

| 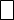 None | 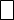 1-4 cases | 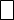 5-10 cases | 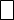 >10 cases |
| --- | --- | --- | --- |

1. ***Stroke (knowledge, identification & assessment):***

- Mention the types of strokes?

|  |  |
| --- | --- |

- Mention 3 cardinal symptoms that patient with acute stroke can have?

|  |  |  |
| --- | --- | --- |
|  |  |  |

- Do you know any scale for acute stroke identification in pre-hospital phase? If yes mention one scale.

|  |  |
| --- | --- |

- Mention 3 mimics of acute stroke ( i.e.; diseases may have similar presentation like acute stroke)?

1-

2-

3-

1. ***Pre-hospital management for stroke patient:***

- Which category you will triage patient with acute stroke?

| - Immediate | - Urgent |
| --- | --- |
| - Less urgent | - Non urgent |

- What you should ask specifically the patient or the family at the scene? (mention 3)

|  |  |  |
| --- | --- | --- |

- Mention 5 steps need to be done for patient with acute stroke?

|  |  |  |
| --- | --- | --- |
|  |  |  |

- Where you should take patient with stroke?
- Nearest hospital– without acute stroke services
- Nearest hospital with stoke unite.
- As per patient or family request
- Do you notifying the hospital for stroke patient arrival? (per-arrival notification)

| - Yes | - No |
| --- | --- |

1. ***Thrombolytic therapy-TPA:***

- Are you aware about thrombolytic therapy?

| - Yes | - No |
| --- | --- |

- If yes; what it is the window period for thrombolytic therapy? ----------
- Mention 4 contraindications for thrombolytic therapy?

|  |  |
| --- | --- |
|  |  |

- What is the major complication of using for thrombolytic therapy?
