# Supplementary material for: Stroke awareness among Dubai emergency medical service staff and impact of an educational intervention
Source: BMC Res Notes. 2017 Jul 6;10:255. doi: 10.1186/s13104-017-2585-x (PMC5500952; doi:10.1186/s13104-017-2585-x)
Supplement: Supplementary file 2 — Additional file 2. Key to the Data collection Form. The answer key which was pre-prepared for marking the pre and post test forms. [file 13104_2017_2585_MOESM2_ESM.docx]

**Key to Questionnaire**

Stroke (knowledge, identification & assessment):

• Mention types of stroke?

Answer: Stroke divided in to: 1.ischemic (thrombotic or embolic**) (2 points)**

2. Hemorrhagic

•Mention 3 cardinal symptoms that patient with stroke can have?

Answer: The cardinal signs of stroke include: (any point of these) **(3points)**

1. Limb Weakness
2. Facial droop
3. Speech impairment
4. vision impairment
5. Headache
6. Dizziness

• Do you know any scale for stroke identification? If yes mention one scale.

Answer: 1. Cincinnati Pre-hospital Stroke Scale **(1point)**

or

2. Los Angeles Pre-hospital Stroke Screen

Or

3. FAST

•Mention 3 stroke mimics?

1. stroke mimics are: (any point of these) **(3points)**

- Hypertensive encephalopathy
- Hypoglycemia
- Migraine
- Seizures
- Conversion disorder
- Systemic infection
- Brain tumor
- Brain abscess

Pre-hospital management for stroke patient:

• Which category you will triage patient with acute stroke? **(1point)**

 Immediate

 Urgent

 Less urgent

 Non urgent

Answer: Immediate

•What you should ask the patient or the family at the scene? Mention 3 **(3points)**

Answer: (any point of these)

1. the Onset of symptoms
2. Past medical history
3. Medications
4. Risk factors

•Mention 5 steps need to be done for patient with acute stroke?

Answer: (any point of these) **(5points)**

1. Manage ABCs
2. Cardiac monitoring
3. Intravenous access
4. Oxygen (as required O2 saturation >92%)
5. Assess for hypoglycemia
6. Keep the patient NPO
7. Alert receiving ED
8. Rapid transport to closest appropriate facility capable of treating acute stroke

•Where you should take patient with stroke? **(1point)**

- Nearest hospital
- Appropriate hospital with stoke unite.
- Depend on patient or family request.

Answer: Appropriate hospital with stoke unite.

•Are you notifying the hospital for stroke patient arrival? **(1point)**

- Yes
- No

Answer: you have to notify the hospital for stroke patient arrival

Thrombolytic therapy-TPA:

•Are you aware about TPA? **(1point)**

- Yes
- No

•What is the window period for thrombolytic therapy? **(1point)**

Answer: less than 4.5 hours from onset of symptoms.

•Mention 4 contraindications for TPA? **(4points)**

Answer: (any point of these)

1. History of intracranial hemorrhage
2. Known AV malformation, neoplasm, or aneurysm.
3. Minor or rapidly improving stroke symptoms
4. Active internal bleeding or acute trauma, such as a fracture
5. Intraspinal surgery, serious head trauma, or previous stroke within the past 3 months
6. Uncontrolled hypertension based on repeated measurements of > 185 mm Hg systolic pressure or > 110 mm Hg diastolic pressure
7. Witnessed seizure at stroke onset

•What is the major complication of using TPA? **(1point)**

Answer: the major complication is bleeding.

**Total points are 27**
